# The Effect of a Product Placement Intervention on Pupil’s Food and Drink Purchases in Two Secondary Schools: An Exploratory Study

**DOI:** 10.3390/nu14132626

**Published:** 2022-06-24

**Authors:** Suzanne Spence, John N. S. Matthews, Lorraine McSweeney, Ashley J. Adamson, Jennifer Bradley

**Affiliations:** 1 Human Nutrition Research Centre, Population Health Sciences Institute, Faculty of Medical Sciences, Newcastle University, Newcastle upon Tyne NE2 4HH, UK; lorraine.mcsweeney@ncl.ac.uk (L.M.); ashley.adamson@ncl.ac.uk (A.J.A.); jen.bradley@ncl.ac.uk (J.B.); 2 Fuse, The Centre for Translational Research in Public Health, Newcastle University, Newcastle upon Tyne NE2 4HH, UK; 3 School of Mathematics, Statistics and Physics, Newcastle University, Newcastle upon Tyne NE1 7RU, UK; john.matthews@ncl.ac.uk

**Keywords:** children, food choice, product placement, secondary school, nudge interventions

## Abstract

Limited research exists on the effectiveness of product placement in secondary schools. We explored the impact of re-positioning sweet-baked goods, fruit, sugar-sweetened beverages (SSBs) and water on pupil’s lunchtime purchases in two secondary schools in North-East England. We employed a stepped-wedge design with two clusters and four time periods. The intervention(s) involved re-positioning selected food and drinks to increase and decrease accessibility of ‘healthier’ and ‘less healthy’ items, respectively. Unidentifiable smartcard data measured the change in number of pupil’s purchasing the above items. McNemar tests were undertaken on paired nominal data in Stata(v15). In School A, pupils purchasing fruit pots from control to intervention increased (*n* = 0 cf. *n* = 81; OR 0, 95% CI 0 to 0.04); post-intervention, this was not maintained. In School B, from control to intervention pupil’s purchasing sweet-baked goods decreased (*n* = 183 cf. *n* = 147; OR 1.2, 95% CI 1 to 1.6). This continued post-intervention (*n* = 161 cf. *n* = 122; OR 1.3, 95% CI 1.0 to 1.7) and was similar for SSBs (*n* = 180 cf. *n* = 79; OR 2.3, 95% CI 1.7 to 3.0). We found no evidence of other changes. There is some evidence that product placement may positively affect pupil’s food and drink purchases. However, there are additional aspects to consider, such as, product availability, engaging canteen staff and the individual school context.

## 1. Introduction

Non-communicable diseases (NCDs) continue to be a cause of excess deaths [1,2]. In the United Kingdom (UK), a third of pupils starting secondary school are already overweight or obese, and the number of children and young people diagnosed with Type 2 diabetes has increased [3,4]. An unhealthy diet is a key factor in the development of NCDs, an unhealthy bodyweight and Type 2 diabetes [1,2]. Therefore, improving dietary intake in children and young people is essential to mitigate the development of NCDs. Children and young people in the UK generally consume excess free sugars and saturated fat, and not enough micronutrients, or fruit and vegetables [5]. To improve the food and drink choices of children and young people, interventions are required to change the environments they interact with and in which they make these choices [6]. Potential interventions can include providing information, guiding choice through a variety of mechanisms, and restricting or eliminating choice [7]. The school environment is an opportune setting to influence pupil’s dietary intakes [8,9]. In 2016, in the UK, there were approximately 3.2 million pupils attending a state-funded UK secondary school [10], highlighting the extent of potential opportunity to influence food and drink choices. Although there have been substantial changes to school food in the UK, including national food standards, which restrict what can and cannot be offered [11], pupils in secondary schools are presented with a variety of food and drinks on a daily basis. Prior research evaluating the impact of food and nutrient based standards on 11–12 year olds’ diet showed limited effects [12].

There is increasing attention in public health on creating environments that make ‘healthier’ eating behaviours easier to adopt at a population-level; one such approach is product placement interventions [13,14]. The intention of a product placement intervention is to influence the selection, purchasing or consumption behaviour of food and drink choices by altering the position or availability [13]. In practice, this requires small changes to the layout of food and drinks, for example, re-positioning water so that it is more accessible than sugar-sweetened beverages (SSBs). These are commonly referred to as ‘nudge interventions’. A feature of such interventions is that individuals are not prohibited from choosing certain foods or drinks [15]. The financial cost of nudge interventions is generally minimal. While school canteens have been part of the focus to implement these interventions, findings from systematic reviews about effectiveness across various settings are inconsistent [16]. There is limited evidence on the use of these interventions in UK secondary schools, current research has mainly been undertaken in the USA. In addition, most studies have focused on fruit and vegetable interventions [17]; and few studies have focused on the effect of product placement with children and young people [18]. In this study, the focus was on re-positioning sweet baked goods, fruit, SSBs, and water during the lunch period in two secondary schools in North East (NE) England. The aim was to re-position these selected food and drinks to increase the accessibility of ‘healthier’ items and decrease the accessibility of ‘less healthy’ items. We subsequently explored the effect of this intervention on the number of pupil’s purchasing these items at lunch time.

## 2. Materials and Methods

### 2.1. Setting and Sample

Using a convenience sample of Head teachers and/or school leads for Food and Nutrition from four secondary schools, study details were emailed, followed by a phone call. Two secondary schools agreed to participate, one declined and one did not respond despite several attempts. School A and B were located in similar socio-economic areas determined at the school level (school level Index of Multiple Deprivation was 5 and 4, respectively; these schools are located in more deprived areas). The schools had the same catering provider. The sample size was determined by the number of pupils in each school receiving a school meal. Each school received a £50 voucher as token of thanks for their participation.

### 2.2. Study Design

We employed a stepped-wedge design consisting of two clusters (school A and B) and four time periods (T1–T4). Each time period included three weeks (15 days) to reflect the menu cycle. Table 1 shows the time periods (control, intervention and post-intervention) by school. As only two schools participated, a pragmatic decision was made that school A would receive the intervention first.

### 2.3. School Interventions

**Figure 1:**
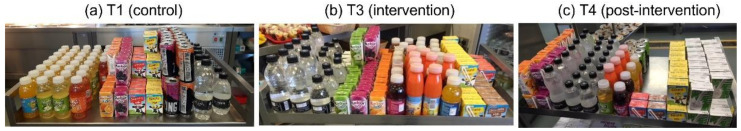
An example of the sugar-sweetened beverage intervention by time period (school A); (**a**) T1 (control) no intervention had taken place (**b**) T3 (intervention) drinks were re-positioned in order of sugar content; least sugar content on the left (i.e., water) to most sugar content on the right (i.e., fruit juice from concentrate and flavoured milks (**c**) T4 (post-intervention).

**Figure 2:**
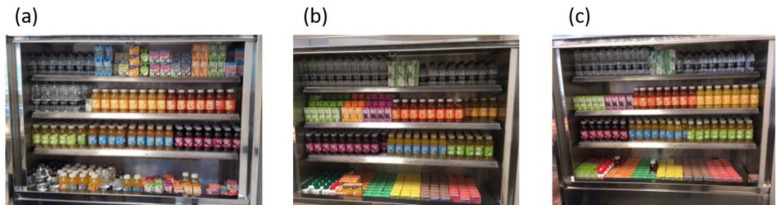
An example of the sugar-sweetened beverage intervention by time period (school B); (**a**) T1 and T2 (control) no intervention had taken place (**b**) T3 (intervention) drinks were re-positioned in order of sugar content; least sugar content at the top (i.e., water & plain milk) to most sugar content at the bottom (i.e., fruit juice from concentrate and flavoured milks (**c**) T4 (post-intervention).

Initial school visits observed the ‘typical’ layout of foods and drinks. During the control period no changes were made to the layout of foods and drinks. During the intervention periods, the intervention(s) focused on the re-positioning of sweet baked goods, fruit, SSBs, and water. The purpose was to increase the accessibility of ‘healthier’ items and decrease the accessibility of ‘less healthy’ items. Fruit was re-positioned in front of the sweet baked goods, and drinks were re-positioned according to sugar content. In school A, water and low SSBs were re-positioned to the left and SSBs with most sugar content on the right, as this was most visible for pupils in the queue (Figure 1). In school B, water and low SSBs were re-positioned to the top of the drink’s cabinet (most visible for pupils in the queue) and high SSBs were re-positioned to the lower shelves (Figure 2). To mitigate non-compliance by schools the intervention(s) were co-produced and agreed with catering staff, and the school lead. All school catering staff received training on where products were to be positioned and the reason for this. Catering staff were given the opportunity to ask questions and clarify issues during this session. The intervention was in place during the lunch period of service only.

To explore fidelity across time periods, photographs were taken. In both schools, during the intervention periods frequent visits were made by the research team to ensure compliance with re-positioning of products; if these were not adhered to the researchers reminded staff and made changes. During time period 4, if products were not re-positioned correctly the researchers did not make changes, this was to explore adherence post-intervention. Informal discussions were held with catering staff about the study, the intervention(s) and the importance of product re-positioning at the start of the study and throughout. In addition, catering staff were involved with discussions about the extra payment buttons that had to be added to the tills for recording pupil purchasing during the study period, all catering staff that used the tills were given the same instructions.

### 2.4. Data Collection

Non-identifiable pupil’s food and drink purchasing data were collected from the smartcard system which captures point of sales data. This includes information on food and drink items purchased, time and date of purchase and, whether purchased items were free (pupils eligible for free school meals) or paid. Pupil’s food and drink purchasing data were collected weekly for the duration of the study during the four time periods. In total 12 weeks (60 days) of food and drink purchasing data were collected. Collaboration with an external company responsible for the smartcard systems in both schools was required to enable data collection, and to ensure data were amenable to data manipulation. A number of changes were made to the till buttons to ensure the detail of purchasing data required for the statistical analysis was captured. For example, if a pupil purchased a ‘wrap & drink’ the option was changed on the till to include the type of drink purchased, i.e., ‘wrap & milk’, ‘wrap & water’ or ‘wrap & juice’.

### 2.5. Ethics, Access to Data and Confidentiality

The study was conducted in accordance with the Declaration of Helsinki and approved by the Faculty of Medical Sciences Ethics Committee of Newcastle University (application No. 1282/14807/2017, date of approval: 10 July 2017).

Data access was restricted to the research team; data processing and storage were managed according to University policies.

### 2.6. Main Outcome Measures

The main outcome measure to assess the effect of the intervention(s) on pupil purchasing was a change in the number of pupils purchasing the selected food and drink categories, that is fruit pots, fruit, yoghurt, sweet baked goods (SSBs), water and Zing (a, SSB) across time periods (T1–T4).

### 2.7. Data Manipulation and Statistical Analysis

Food and drink purchases by a pupil were obtained for each day in each week of the study. These data were then linked by a unique pupil identifier for the 12 weeks in both schools. Pupil food and drink purchases were grouped at two levels. Firstly, food and drink items were categorised to create groups for data analysis (for example, sweet baked goods included: cakes, muffins and tray bakes). Although Zing is an SSB, this item was grouped separately for analysis, this was because School A removed the sale of Zing in their school during T3 (the second intervention time period) as catering staff became aware of the sugar content. Only food and drinks purchased at lunchtime (between 12:15 p.m. and 13:00 p.m.) were included in the analysis. Secondly, data was grouped as total sales to include all time periods (i.e., T1–T4 combined) and then by individual time periods (i.e., T1, T2, T3 and T4).

The first analysis provided a simple description of the total sales of food and drink items purchased at lunchtime by school. The second analysis provided a more detailed examination on the effect of the intervention(s) on pupil purchasing at lunchtime by school. The analyses classified each pupil as a purchaser if they made any purchases in the period of the design, and therefore does not explore the effect on the number of purchases made by a pupil for each item.

McNemar tests were undertaken on paired nominal data only for those food and drink items included in the intervention(s), for example, sweet baked goods and SSBs (Table 2: example of McNemar calculation). All analyses were conducted in Stata version 15 (StataCorp. 2017. College Station, LLC, Los Angeles, CA, USA).

## 3. Results

Non-identifiable purchasing data were collected from pupils in school A (*n* = 540) and school B (*n* = 1194). The number of pupils that had a free (pupils eligible for a free school meal) or paid meal by school were: school A free school meal *n* = 258 (48%), paid school meal *n* = 282 (52%); school B free school meal *n* = 433 (36%), paid school meal *n* = 761 (64%).

### 3.1. Total Sales of the Top Ten Pupil Purchases by School

**Figure 3:**
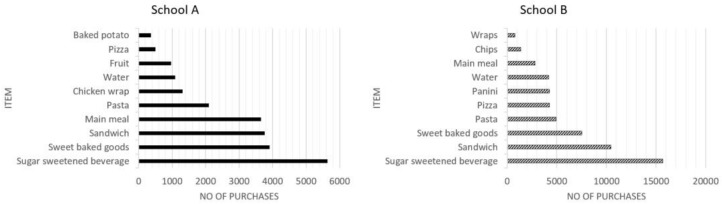
Top ten food and drink purchases at lunchtime by school (total sales data).

The top ten pupil purchases at lunchtime are shown in Figure 3; this is the total sales from all time periods (T1 to T4). In both schools, SSBs were the main purchase by pupils, followed by items such as sweet baked goods, sandwiches, pasta, pizza slices, paninis and wraps, which are mainly ‘grab and go’ items. The ‘main meal’ option was a popular purchase in school A (ranked 4th) but was less popular in school B (ranked 8th).

### 3.2. Effect of the Intervention(s) on Pupil Purchasing by School

In school A, we found no evidence of an intervention(s) effect on pupil purchasing for water, SSBs, yoghurt and sweet-baked goods across any of the time periods (Table 3). We found some limited evidence of an effect of the intervention(s) on pupil purchasing for Zing (an SSB) and fruit pots. The number of pupils that purchased Zing from the control to the intervention period decreased (*n* = 85 cf. *n* = 9; Odds Ratio (OR) 9.4, 95% confidence interval (CI) 4.7 to 21.4). The number of pupils that purchased fruit pots from the control (T1) to the intervention (T2) period increased (*n* = 0 cf. *n* = 81; OR 0, 95% CI 0 to 0.04). We found evidence of a decrease in the number of pupils that purchased fruit in the intervention period (*n* = 48 cf. *n* = 26; OR 1.8, 1.1 to 3.1). During T2 and T3 (both intervention periods), there was evidence of a continued decrease on the number of pupils purchasing Zing (Table 3). There was no evidence of a difference in the number of pupils purchasing fruit pots, i.e., the change was maintained. The number of pupils that purchased fruit decreased (*n* = 39 cf. *n* = 9; OR 4.3, 2.1 to 10.2). Post-intervention, the increase in fruit pot purchases was not maintained; the number of pupils that purchased fruit pots decreased between T3 and T4 (*n* = 81 cf. *n* = 6; OR 13.5, 5.9 to 37.9). In contrast, the number of pupils that purchased fruit increased (*n* = 12 cf. *n* = 47; OR 0.3, 0.1 to 0.5).

In school B, we found no evidence of an effect of the intervention(s) on pupil purchasing for yoghurt or fruit across any of the time periods (Table 4). During the control (T2) and intervention period (T3) there was no evidence that the intervention(s) had an effect on pupil purchasing for water, SSBs, fruit pots or yoghurt. During this period, the number of pupils that purchased sweet baked goods decreased (*n* = 183 cf. *n* = 147; OR 1.2, 1 to 1.6). Between T3 (intervention) and T4 (post-intervention) the number of pupils that purchased sweet baked goods continued to decrease (*n* = 161 cf. *n* = 122; OR 1.3, 1.0 to 1.7), this was similar for SSBs (*n* = 180 cf. *n* = 79; OR 2.3, 1.7 to 3.0). The number of pupils that purchased fruit pots or water decreased, and this was statistically significant (Table 4).

## 4. Discussion

### 4.1. Summary of Key Results

In both schools, SSBs were the main purchase by pupils at lunchtime, followed by ‘grab and go’ items. There was some limited evidence that the intervention(s) had an effect on pupil purchasing in both schools, but the effects were inconsistent.

In school A, during T1 and T2 the number of pupils purchasing the SSB Zing decreased. However, between T2 and T3, catering staff became aware of the sugar content in Zing due to the intervention and removed Zing from sales explaining the statistically significant decrease. There was no effect in school A on pupils purchasing SSB post-intervention. In contrast, in school B, there was a statistically significant decrease in the number of pupils purchasing an SSB post-intervention. In school A, we found no evidence that re-positioning sweet baked goods affected pupil purchases. In comparison, in school B, there was evidence of a decrease in pupils purchasing sweet-baked goods post-intervention.

In school A, during the intervention the number of pupils purchasing fruit pots increased, although the effect was not maintained post-intervention; this was similar for school B. In school A, while fruit pot purchases increased during the intervention period, purchases of whole fruit decreased, this may be explained by pupils switching to purchase fruit pots rather than the whole fruit on offer. Observations in school A during the post-intervention period indicated fewer fruit pots were available to purchase, therefore, the availability, or lack of, fruit pots may explain these findings, and the potential switching between purchasing fruit pots and whole fruit. The difference in findings by school may be due to factors such as the space available to implement changes to product layout and catering staff compliance.

### 4.2. Relationship to Other Studies

There is evidence from studies that nudge interventions, including product placement in different settings have positive effects. Ensaff et al., 2015 explored the use of a combination of small changes (i.e., product placement, posters and labelling) on pupil’s selection of fruit and vegetable items in one intervention and one control secondary school in England. Overall, they found positive changes to pupil’s choices during and post-intervention periods, for example, pupils were 2.5 times more likely to select fruit and vegetable items during the intervention period, compared to baseline [19]. Studies in school canteens in the USA report positive effects on pupil’s food choices by increasing convenience, and product placement [20,21,22]. In a meta-analysis of nudge interventions using product placement to increase fruit and/or vegetable choice, sales or servings, findings highlighted positive effects [16]. Whilst we found evidence of increased pupil purchasing for fruit pots, it is difficult to fully associate this to the product placement intervention due to product availability fluctuating over the time periods, this is similar for findings on pupil purchases of Zing.

A study by van Kleef et al. 2012 [15] explored the effect of shelf arrangement (i.e., accessibility) and assortment structure (i.e., availability) of healthy and unhealthy snacks on shelves in a hospital canteen and collected daily sales data. They found that increased availability of healthy snacks resulted in increased sales [15]. Similarly, in school A when fruit pots were available there was a statistically significant increase in pupil purchasing, but this was not consistent in school B. Shifting the focus to interventions that are centred around product availability in schools may be more effective in improving pupil’s dietary intakes. Van Kleef et al. 2012 [15] and Shepherd 2002 [23] discuss the role ‘habit’ plays in food and drink choices; these findings support the view by Shepherd 2002 the modification of dietary habits is a gradual process [23]. This was inadvertently expressed by pupils in the qualitative component to this work, pupils discussed purchasing the same food and drink items daily as they knew these were always available, therefore, they did not take time at the counter to consider all options [24].

### 4.3. Strengths and Limitations

This is one of the first UK studies to explore the effect of re-positioning SSBs, sweet-baked goods and fruit on pupil purchases using a stepped-wedge study design. The use of the stepped-wedge design was advantageous for evaluating population-level effects and examining with-in school intervention effects [25], however, there were limitations associated with this design, noted below. Few studies have included a post-intervention period, this allowed us to explore the intervention effects when schools had autonomy to continue with the intervention, or not. Researchers did not alter product layout during this period. This study focused beyond fruit and vegetable interventions to include sweet baked goods and SSBs, and so, contributing to the evidence-base on the use of nudge interventions to improve children’s diets. Smart card data provided a large amount of unidentifiable pupil purchasing data and enabled us to collect data without influencing pupil food and drink choices. The findings reported include effect sizes of the intervention by time periods and number of pupils—in the systematic review and meta-analysis by Broers et al., 2017 they noted this is often omitted in reporting [16].

There are notable limitations in this study. The number of food and drink items on offer per day may have fluctuated, the space available for product placement varied by school, and compliance with the interventions potentially varied by staff. Within the school-based setting a major limitation with these study designs is the inability to control for all conditions, so for example, as mentioned the display space available, and as in this study, staff became aware of the sugar content of Zing and removed this product, we therefore separated Zing from the analysis of SSBs to ensure analysis was not affected. Whilst we employed a stepped-wedge design, the analysis does not consider the between school effect, results are reported separately by school. It became evident during the study that product placement was impacted at a school-level due to practical aspects such as space available, i.e., layout was different, therefore, these analyses only considered the within-school affect across time periods. Furthermore, this study did not record the daily number of items on sale as the focus was on product placement, nor did the study record the number of times schools did not comply as changes were made prior to pupil purchases, however this would not have been captured on non-visit days.

It is also important to highlight that these data reflect pupil purchases and not consumption. This study only explored the effect of a product placement intervention on selected food and drink items; we have not explored the potential wider implications on other food choices by pupils. The analyses do not consider the effect of gender or age due to the non-identifiable data used. This was an exploratory study and conducted in only two schools in NE England, therefore the generalisability is limited.

### 4.4. Future Research and Implications for Policy and Practice

This was an exploratory study to explore the use of product placement in a real-life school canteen setting. This study has highlighted several practical limitations for consideration in future large-scale studies to develop the evidence-base of these interventions in schools. Consideration of the methodological and analytical challenges is needed to improve and evaluate nudge interventions, including product placement and availability. Whilst the focus of this intervention covered the lunch-time period, pupil smartcard data includes purchases across the school day. Anecdotal evidence from these data suggests break-time purchases require improvement and future interventions may need to include the whole school day to positively influence pupil purchasing. In accompanying qualitative work, pupils discussed that a cookie was standard in a meal deal, but to substitute this with fruit costs more, and they were not willing to pay the extra [24]. Future interventions may also consider the effect of pricing implications in schools. Involving pupils in co-designing future interventions may also have a beneficial influence on their food and drink choices [26,27].

Improving pupil’s diets is a public health priority, however central to the development of school-based interventions is the need to be pragmatic for schools to implement and to avoid unintended consequences. School canteens are busy environments and the issue of queuing at lunchtime has been well-documented [28]. The lunchtime period in these schools was approximately 45 min, which is not unique to this study. During this time canteen staff serve large numbers of pupils. Designing interventions requires balancing what is feasible, and consideration of the nutritional detail required to measure outcomes to avoid burdening canteen staff and negatively impacting queuing. Collaboration and engagement with canteen staff in the intervention design, rationale, feasibility, and importance of compliance and consistency in recording are key to success in implementation of school-based interventions.

This study provides some limited evidence that implementing these interventions may positively affect pupil purchasing. Within the limitations of this study, these findings highlight the issue of product availability in secondary schools. Along with compliance of school food-based standards, product availability in secondary schools must be addressed to improve pupil’s food choices and therefore, dietary intake.

## Figures and Tables

**Table 1:** Time periods by school.

	Time Period			
School	T1	T2	T3	T4
School A	C *	I ^†^	I	PI ^‡^
School B	C	C	I	PI

* Control; ^†^ Intervention; ^‡^ Post-intervention.

**Table 2:** Example of McNemar calculation.

	T1		
T2		No	Yes
	No	a *	b ^†,ǁ^
	Yes	c ^ǂ,ǁ^	d ^§^

* total number of pupils that did not purchase in either T1 or T2; ^†^ total number of pupils that purchased in T1 but not T2; ^ǂ^ total number of pupils that purchased in T2 but not T1; ^§^ total number of pupils that purchased in T1 and T2; ^ǁ^ cells are used in the McNemar Test: x^2^ = (b − c)^2^/(b + c) on 1 degree of freedom.

**Table 3:** School A: Effect of intervention on pupil’s food and drink purchases across time periods with OR, 95% CI and *p*-value.

	School A (Total Number of Pupils *n* = 540)											
	T1 *&T2 ^†^				T2&T3 ^‡^				T3&T4 ^§^			
Item	D1 ^ǁ^	D2 ^¶^	OR (95% CI)	*p*-Value	D3 **	D4 ^††^	OR (95% CI)	*p*-Value	D5 ^‡‡^	D6 ^§§^	OR (95% CI)	*p*-Value
	*n*	*n*			*n*	*n*			*n*	*n*		
Water	60	62	1.0 (0.7 to 1.4)	0.9	66	76	0.9 (0.6 to 1.2)	0.5	78	61	1.3 (0.9 to 1.8)	0.2
SSBs	50	59	0.8 (0.6 to 1.3)	0.4	61	73	0.8 (0.6 to 1.2)	0.3	67	48	1.4 (0.9 to 2.1)	0.09
Zing	85	9	9.4 (4.7 to 21.4)	<0.001	79	0	0 (21 to 0)	<0.001	0	0	-	-
Yoghurt	3	1	3 (0.2 to 157.5)	0.6	1	1	1 (0.01 to 78.5)	1.0	1	2	0.5 (0.0 to 9.6)	1.0
Sweet baked goods	62	69	0.9 (0.6 to 1.3)	0.5	71	68	1 (0.7 to 1.5)	0.9	57	68	0.8 (0.6 to 1.2)	0.4
Fruit Pots	0	81	0 (0 to 0.04)	<0.001	41	53	0.8 (0.5 to 1.2)	0.3	81	6	13.5 (5.9 to 37.9)	<0.001
Fruit	48	26	1.8 (1.1 to 3.1)	0.01	39	9	4.3 (2.1 to 10.2)	<0.001	12	47	0.3 (0.1 to 0.5)	<0.001

* T1 (control) ^†^ T2 (intervention) ^‡^ T3 (intervention) and ^§^ T4 (post-intervention); **^ǁ^** D1 (*n* = pupils purchasing in T1 & not in T2); ^¶^ D2 (*n* = pupils purchasing in T2 & not in T1) ** D3 (*n* = pupils purchasing in T2 & not in T3); **^††^** D4 (*n* = pupils purchasing in T3 & not in T2); **^‡‡^** D5 (*n* = pupils purchasing in T3 & not in T4); ^§§^ D6 (*n* = pupils purchasing in T4 & not in T3).

**Table 4:** School B: Effect of intervention on pupil’s food and drink purchases across time periods with OR, 95% CI and *p*-value.

	School B (Total Number of Pupils *n* = 1194)											
	T1 *&T2 ^†^				T2&T3 ^‡^				T3&T4 ^§^			
Item	D1 ^ǁ^	D2 ^¶^	OR (95% CI)	*p*-Value	D3 **	D4 ^††^	OR (95% CI)	*p*-Value	D5 ^‡‡^	D6 ^§§^	OR (95% CI)	*p*-Value
	*n*	*n*			*n*	*n*			*n*	*n*		
Water	130	146	0.9 (0.7 to 1.1)	0.4	144	163	0.9 (0.7 to 1.1)	0.3	193	98	2.0 (1.5 to 2.5)	<0.001
SSBs	123	150	0.8 (0.6 to 1.0)	0.1	158	161	1.0 (0.8 to 1.2)	0.9	180	79	2.3 (1.7 to 3.0)	<0.001
Zing ^¶¶^	-	-	-	-	-	-	-	-	-	-	-	-
Yoghurt	2	8	0.3 (0.03 to 1.3)	0.06	9	5	1.8 (0.5 to 6.8)	0.3	5	4	1.3 (0.3 to 6.3)	0.7
Sweet baked goods	175	131	1.3 (1.0 to 1.7)	0.01	183	147	1.2 (1.0 to 1.6)	0.05	161	122	1.3 (1.0 to 1.7)	0.02
Fruit Pots	54	24	2.3 (1.4 to 3.8)	<0.001	30	34	0.9 (0.5 to 1.5)	0.6	40	21	1.9 (1.1 to 3.4)	0.01
Fruit	12	11	1.1 (0.4 to 2.7)	0.8	12	22	0.5 (0.2 to 1.2)	0.08	20	12	1.7 (0.8 to 3.7)	0.2

* T1 (control) ^†^ T2 (intervention) ^‡^ T3 (intervention) and ^§^ T4 (post-intervention); ^ǁ^ D1 (*n* = pupils purchasing in T1 & not in T2); ^¶^ D2 (*n* = pupils purchasing in T2 & not in T1) ** D3 (*n* = pupils purchasing in T2 & not in T3); **^††^** D4 (*n* = pupils purchasing in T3 & not in T2); **^‡‡^** D5 (*n* = pupils purchasing in T3 & not in T4); ^§§^ D6 *(n* = pupils purchasing in T4 & not in T3); ^¶¶^ Zing (not available in school B).

## Data Availability

Not applicable.
